# Comparison of Goal-Directed Fluid Therapy and Conventional Fluid Therapy in Elective Major Abdominal Surgery: A Meta-Analysis of Randomized Controlled Trials

**DOI:** 10.7759/cureus.110243

**Published:** 2026-06-04

**Authors:** Nashrah Ashraf, Owais ul Umer Zargar, Aayat Albina

**Affiliations:** 1 Anesthesiology, Acharya Shri Chander College of Medical Sciences and Hospital, Jammu, IND; 2 General Surgery, Acharya Shri Chander College of Medical Sciences and Hospital, Jammu, IND; 3 Obstetrics and Gynecology, Acharya Shri Chander College of Medical Sciences and Hospital, Jammu, IND

**Keywords:** conventional fluid therapy, elective abdominal surgery, gastrointestinal recovery, goal-directed fluid therapy, hospital length of stay, major abdominal surgery, meta-analysis, postoperative ileus, postoperative morbidity, randomized controlled trials

## Abstract

Goal-directed fluid therapy (GDFT) has emerged as an important perioperative strategy aimed at optimizing hemodynamic status and improving surgical outcomes; however, evidence regarding its effectiveness in patients undergoing major abdominal surgery remains variable. This meta-analysis was conducted to evaluate the impact of intraoperative GDFT compared with conventional fluid therapy on postoperative morbidity and clinical outcomes in adult patients undergoing elective major abdominal surgery. Randomized controlled trials comparing GDFT with conventional intraoperative fluid therapy were analyzed. The assessed outcomes included postoperative morbidity, 30-day mortality, length of hospital stay, intensive care unit (ICU) stay, recovery of gastrointestinal function, and incidence of paralytic ileus. A total of 14 randomized controlled trials involving approximately 2,750 patients were included, with 1,433 patients receiving GDFT and 1,317 receiving conventional fluid therapy. GDFT was associated with a significant reduction in hospital length of stay (mean difference (MD) 2.5 days, 95% confidence interval (CI) 4.5 to -0.5), earlier passage of flatus (MD 6.8 hours, 95% CI 11.2 to -2.4), earlier tolerance of oral intake (MD 15.2 hours, 95% CI 26.8 to -3.6), and reduced incidence of postoperative ileus (risk ratio (RR) 0.48, 95% CI 0.28-0.82). However, no significant reduction was observed in overall postoperative morbidity (RR 0.78, 95% CI 0.57-1.07) or mortality (RR 1.02, 95% CI 0.45-2.34). Intraoperative GDFT in elective major abdominal surgery was associated with improved postoperative gastrointestinal recovery, reduced incidence of postoperative ileus, and shorter hospital length of stay compared with conventional fluid therapy. However, no significant reduction in overall postoperative morbidity or mortality was observed. These findings support the use of GDFT as a safe and effective perioperative strategy to enhance postoperative recovery in major abdominal surgery.

## Introduction and background

Intraoperative hypovolemia resulting from the loss of as little as 10-15% of circulating blood volume can significantly reduce splanchnic perfusion [1], and this reduction may persist even after the hypovolemic state has resolved [2]. This decreased perfusion leads to intramucosal acidosis of the gastrointestinal tract, triggering a cascade of physiological events that impair postoperative gastrointestinal function and contribute to complications [2-4]. Postoperative gastrointestinal morbidity, manifesting as intolerance to oral or enteral feeding, nausea, vomiting, and abdominal distension, accounts for more than half of delayed hospital discharges. Recognition of this problem led to the development of intraoperative goal-directed fluid therapy (GDFT). In this approach, small boluses of fluid (approximately 200-250 mL), usually colloids, are administered over a background crystalloid infusion to increase stroke volume and cardiac output, enhance gut perfusion, and reduce intestinal mucosal acidosis [3,5,6]. Conventional fluid therapy refers to standard intraoperative fluid management without protocolized, hemodynamic-guided optimization, as specified in the individual study protocol.

Several monitoring techniques have been developed to guide intraoperative fluid therapy by measuring stroke volume and cardiac output. These include transesophageal Doppler (TED), lithium dilution methods, arterial pulse contour analysis, thoracic electrical bioimpedance, partial non-rebreathing systems, and transpulmonary thermodilution techniques [5]. Among these, transesophageal Doppler and lithium dilution methods are the most commonly used in clinical practice. The typical algorithm involves assessing the change in stroke volume following a fluid bolus of 200-250 mL administered over five to 10 minutes [6-8]. An increase in stroke volume of more than 10% indicates hypovolemia, suggesting that an additional fluid bolus is required. Conversely, an increase of 10% or less suggests adequate intravascular volume, and fluid management should continue with the background crystalloid infusion without further boluses. If, during ongoing monitoring, the stroke volume decreases by more than 10%, another fluid bolus is administered, and the assessment cycle is repeated [8,9]. Variations of this protocol include monitoring stroke volume variation (SVV) and corrected flow time (FTc) to further optimize intraoperative fluid management [10-12].

Since its introduction in the early 2000s, GDFT has been evaluated in multiple randomized controlled trials and meta-analyses. These studies demonstrated reductions in postoperative complications and hospital length of stay compared with conventional fluid management strategies [13-16]. The accumulating evidence contributed to the incorporation of GDFT into perioperative care pathways and informed recommendations from the UK National Institute for Health and Clinical Excellence (NICE) [17-20].

However, many of these early studies did not clearly define postoperative fluid management protocols, and perioperative care strategies were often not standardized. Subsequent evidence has shown that avoiding postoperative salt and water overload and maintaining patients in a near-zero fluid balance can independently reduce complication rates and shorten hospital stay, even in patients who do not receive intraoperative GDFT [19-23]. The aim of this meta-analysis of randomized controlled trials comparing intraoperative GDFT with conventional fluid therapy in adult patients undergoing elective major abdominal surgery was to evaluate and compare the effects of these therapies on postoperative complications, length of hospital stay, gastrointestinal recovery, and mortality.

## Review

Methods

A comprehensive literature search was conducted across PubMed, MEDLINE, Web of Science, Google Scholar, and the Cochrane Library to identify studies evaluating the effect of intraoperative GDFT on postoperative outcomes in patients undergoing elective surgery. Studies published in English between January 2013 and December 2025 were considered. The PubMed search strategy included the terms “goal-directed fluid therapy” OR “flow-directed fluid therapy” combined with “surgery” OR “intraoperative.” Equivalent search strategies were adapted for the remaining databases. The reference lists of eligible studies and relevant review articles were manually screened to identify additional studies. The search was limited to adult patients undergoing elective surgical procedures. This meta-analysis was conducted in accordance with the Preferred Reporting Items for Systematic Reviews and Meta-Analyses (PRISMA 2020) guidelines.

Titles and abstracts identified through the search strategy were initially screened, followed by full-text review of potentially eligible studies. Studies were included if they involved adult patients undergoing elective major abdominal surgery, compared GDFT with conventional intraoperative fluid therapy in a randomized design, and reported at least one relevant postoperative outcome. Major abdominal surgery was defined as general, vascular, gynecological, and urological procedures involving bowel manipulation. Studies were excluded if they involved non-abdominal surgeries such as cardiac, orthopedic, or peripheral vascular procedures, included emergency surgeries, failed to report relevant clinical outcomes, or used GDFT in both study arms. Any uncertainties regarding study eligibility were resolved through discussion before final inclusion.

Data extraction was performed by one reviewer (NA) and independently verified by a second reviewer (OZ) to ensure accuracy. The primary outcome evaluated was postoperative morbidity. Postoperative morbidity was defined as the occurrence of one or more postoperative complications reported by the included studies, including surgical site infection, anastomotic leak, pulmonary complications, cardiovascular complications, urinary complications, sepsis, and other clinically significant adverse events occurring during the postoperative period. Secondary outcomes included 30-day mortality, length of hospital stay, ICU stay, time to recovery of gastrointestinal function (including passage of flatus and stool), and incidence of paralytic ileus. Additional data collected included patient demographics (age, sex, and ASA status), surgical characteristics (type of surgery, laparoscopic procedures, and blood loss), and intraoperative fluid management variables (total fluid administration, crystalloids, colloids, and inotrope use). When required data were unavailable, corresponding authors were contacted up to three times over a six-week period. If data remained unavailable, medians and interquartile ranges were converted to means and standard deviations using the method described by Hozo et al., in which the standard deviation was estimated as the upper limit of the interquartile range minus the lower limit divided by 1.35 [12]. The risk of bias for included studies was assessed using the Cochrane Collaboration Risk of Bias tool implemented in Review Manager (RevMan) version 5.3 (The Cochrane Collaboration, Copenhagen). The domains evaluated included random sequence generation, allocation concealment, blinding of participants and personnel, blinding of outcome assessment, incomplete outcome data, and selective outcome reporting.

Statistical analysis was performed using RevMan version 5.3 software. Dichotomous outcomes were expressed as risk ratios (RR) with 95% confidence intervals (CIs) and analyzed using the Mantel-Haenszel random-effects model. Continuous outcomes were expressed as mean differences (MDs) with 95% CI and analyzed using the inverse-variance random-effects model. Forest plots were constructed to present the pooled results, and a two-tailed p-value <0.05 was considered statistically significant. Heterogeneity among studies was assessed using the I² statistic, where values <25% were considered low heterogeneity, 25-50% moderate heterogeneity, and >50% high heterogeneity. The overall quality of evidence for each outcome was evaluated using GRADEpro software.

Results

**Figure 1:**
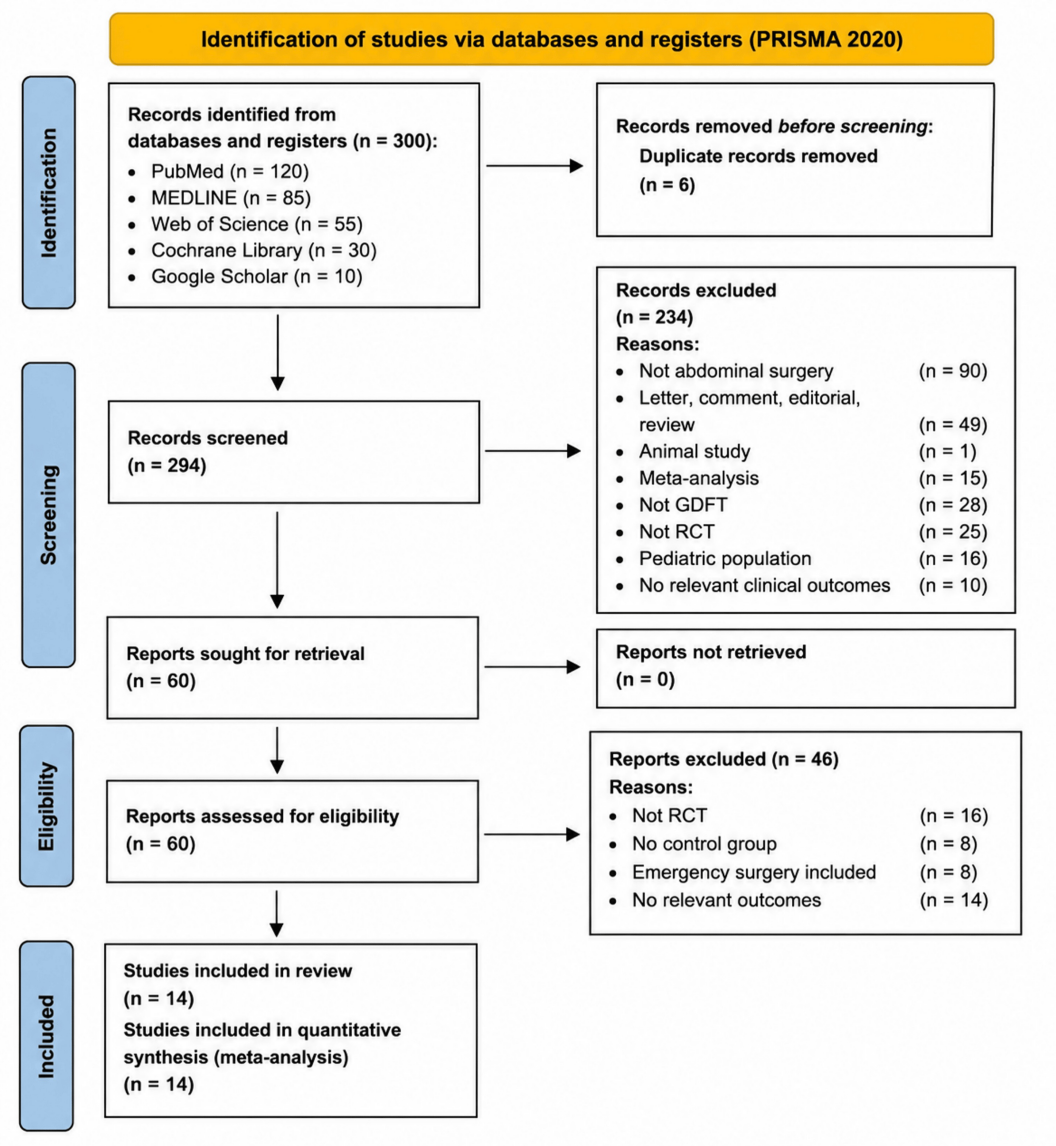
PRISMA flow diagram illustrating the process of identification and selection of relevant studies from the initial database search PRISMA, Preferred Reporting Items for Systematic Reviews and Meta-Analyses; RCT, Randomized controlled trial; GDFT, Goal-directed fluid therapy.

From an initial pool of 300 identified studies, after removing duplicates and screening titles and abstracts, 60 articles were assessed for eligibility. Of these, 46 articles were excluded due to non-relevance, non-randomized design, or inappropriate population, and no relevant outcomes. Finally, 14 randomized controlled trials were included in the meta-analysis (Figure 1) [24-47]. Among these, two studies were conducted in colorectal surgery, two in hepato-pancreaticoduodenal procedures, two in urology, seven encompassed a broad range of abdominal surgeries, and one was in gynecology.

Overall, the included studies demonstrated a low risk of bias and generally high methodological quality. The risk of bias of the included studies was assessed using the Cochrane Risk of Bias tool. Most studies demonstrated a low risk of bias across domains, including random sequence generation, allocation concealment, and completeness of outcome data. However, some studies showed unclear or high risk of bias in blinding of participants and personnel due to the nature of the intervention. Overall, the included studies were judged to have low to moderate risk of bias as summarized in Table 1.

**Table 1:** Risk-of-bias assessment of the included studies

Study	Random sequence generation	Allocation concealment	Blinding (participants/personnel)	Blinding (outcome assessment)	Incomplete outcome data	Selective reporting	Other bias	Overall risk
Arslan-Carlon et al. [30]	Low	Low	Low	Low	Low	Low	Low	Low
Calvo-Vecino et al. [33]	Low	Low	Low	Low	Low	Low	Low	Low
Castro et al. [24]	Low	Unclear	Unclear	Low	Low	Low	Low	Moderate
Coeckelenbergh et al. [41]	Low	Low	Low	Low	Low	Low	Low	Low
de Waal et al. [29]	Low	Low	Low	Low	Low	Low	Low	Low
Hokenek et al. [47]	Low	Unclear	High	Low	Low	Low	Low	Moderate
Yoon et al. [28]	Low	Low	Unclear	Low	Low	Low	Low	Low
Diaper et al. [26]	Low	Low	Unclear	Low	Low	Low	Low	Low
Wu et al. [36]	Low	Low	Unclear	Low	Low	Low	Low	Low
Ramsingh et al. [21]	Low	Unclear	Unclear	Unclear	Low	Low	Low	Moderate
Redondo Calvo et al. [45]	Low	Low	Unclear	Low	Low	Low	Low	Low
Schmid et al. [44]	Low	Low	Unclear	Low	Low	Low	Low	Low
Sujatha et al. [34]	Low	Unclear	High	Low	Low	Low	Low	Moderate
Sun et al. [27]	Low	Low	Low	Low	Low	Low	Low	Low

The quality of evidence for each outcome assessed in the meta-analysis is summarized in Table 2. The overall certainty of evidence ranged from low to moderate to high. The baseline characteristics of studies are shown in Table 3.

**Table 2:** Grade evidence profile RCTs, Randomized controlled trials; GRADE, Grading of Recommendations Assessment, Development and Evaluation.

Outcome	No. of studies	Study design	Risk of bias	Inconsistency	Indirectness	Imprecision	Publication bias	Certainty of evidence
Postoperative complications	10–11	RCTs	Not serious	Not serious	Not serious	Not serious	Undetected	High (⭐⭐⭐⭐)
30-day mortality	4–5	RCTs	Not serious	Not serious	Not serious	Serious	Undetected	Moderate (⭐⭐⭐)
Hospital length of stay (LOS)	8–9	RCTs	Not serious	Serious	Not serious	Serious	Undetected	Low (⭐⭐)
ICU/ITU length of stay	4–5	RCTs	Not serious	Serious	Not serious	Serious	Undetected	Low (⭐⭐)
Postoperative ileus / GI dysfunction	3–5	RCTs	Not serious	Not serious	Not serious	Serious	Undetected	Moderate (⭐⭐⭐)
Time to GI recovery (flatus/oral intake)	3–4	RCTs	Not serious	Not serious	Not serious	Serious	Undetected	Moderate (⭐⭐⭐)

**Table 3:** Baseline characteristics of the included studies GDFT, Goal-directed fluid therapy; Lap, Laparoscopic procedures; ASA, American Society of Anesthesiologists.

Study	GDFT	Control	Surgery	Lap (GDFT)	Lap (Control)	ASA (GDFT)	ASA (control)
Redondo Calvo et al. [45]	19	16	All major liver surgeries	0	0	0:13:6:0	0:10:6:0
Coeckelenbergh et al. [41]	45	45	All major liver surgeries	0	0	Not stated	Not stated
Wu et al. [36]	58	56	Laparoscopic radical resection of colon cancer	58	56	0:25:33:0	0:22:34:0
Castro et al. [24]	43	42	All major bowel surgeries	0	0	7:31:5:0	11:26:5:0
Sun et al. [27]	50	50	Seven gastrectomies, two liver surgeries, four Whipple procedures, 17 hemicholectomies, 12 rectum resections, one radicle cystectomy, three radical resections for gynecological cancer, three other oncological resection procedures, nine stomas	38	31	6:36:8:0	8:33:9:0
Yoon et al. [28]	36	39	Eight open radical cystectomies with ileal conduit, 31 open radical cystectomies with neobladder	0	0	4:29:6:0	2:27:7:0
Hokenek et al. [47]	39	39	Total abdominal hysterectomy with bilateral salpingo-oophorectomy	0	0	Not stated	Not stated
de Waal et al. [29]	274	259	38 oesophageal resection, 97 Whipple, 35 AAA repair, 11 resection of large soft tissue mass, 12 total gastrectomy, 27 colorectal surgery, 14 other abdominal surgery	0	0	24:123:86:1	17:132:95:4
Diaper et al. [26]	200	201	100 visceral, 43 urologic, 53 vascular	0	0	Not stated	Not stated
Arslan-Carlon et al. [30]	142	141	All open radical cystectomy	0	0	Not stated	Not stated
Sujatha et al. [34]	200	101	49 Whipple's procedure, 49 abdominoperineal resection, 55 hemicolectomy, 40 total distal gastrectomy, two GIST excision and intestinal anastomosis, 23 GJ+JJ	0	0	95:105	2 days, 11:42:00
Calvo-Vecino et al. [33]	209	211	150 gastrointestinal, 48 urological, 11 gynecological	105	109	14:122:73:0	35:136:40:0
Weinberg et al. [39]	26	26	All pancreaticoduodenal surgeries	0	0	Not stated	Not stated
Schmid et al. [44]	92	88	16 Whipple, 71 oesophageal, three pancreatectomies, two others	0	0	Not stated	Not stated

Demographics

A total of approximately 2,750 patients were included across the selected studies, with 1,433 (52.1%) patients allocated to the GDFT group and 1,317 (47.9%) to conventional fluid therapy. Within the GDFT arm, fluid management was guided by cardiac output/stroke volume-based systems in 600 patients, oesophageal Doppler in 224 patients, thermodilution in 90 patients, and dynamic preload indices, including stroke volume variation (SVV) in 18 patients, pulse pressure variation (PPV) in 58 patients, pleth variability index (PVI) in 141 patients, and combined SVV/CI or related multimodal parameters in the remaining patients.

Fluid Therapy

There was substantial heterogeneity in intraoperative fluid therapy across the included studies (Table 4). Overall, several studies demonstrated reduced total fluid administration with GDFT compared to conventional therapy. For example, Castro et al. [24] reported lower total fluid volumes in the GDFT group (3810.4 ± 2126.9 mL vs. 4879.9 ± 2147.3 mL), a finding consistent with Redondo et al. [45] (1125.8 ± 751.2 mL vs. 2853.1 ± 1432.2 mL) and Weinberg et al. [39] (2050 (1313-2700) mL vs. 4088 (3400-4525) mL).

**Table 4:** Intraoperative fluid therapy, blood loss, and inotrope use across the included studies Values are presented as mean ± standard deviation or median (interquartile range), as reported in the original studies. Inotrope use is reported as a relative difference between groups where quantitative data were unavailable. Some studies reported infusion rates (mL/kg/h), which were not directly comparable and are therefore not included. NR, Not reported; GDFT, Goal-directed fluid therapy.

Study	Total fluid (mL) GDFT	Total fluid (mL) Control	Crystalloid (mL) GDFT	Crystalloid (mL) Control	Colloid (mL) GDFT	Colloid (mL) Control	Blood loss (mL) GDFT	Blood loss (mL) Control	Inotrope use
Arslan-Carlon et al. [30]	NR	NR	NR	NR	NR	NR	NR	NR	No difference
Koo et al. [37]	NR	NR	NR	NR	NR	NR	NR	NR	Lower in GDFT
Calvo-Vecino et al. [33]	NR	NR	NR	NR	NR	NR	NR	NR	Lower in GDFT
Castro et al. [24]	3810.4 ± 2126.9	4879.9 ± 2147.3	2982.9 ± 1524.0	3901.2 ± 1475.1	779.7 ± 565.1	737.8 ± 383.2	1100.1 ± 851.1	1283.2 ± 959.7	NR
Coeckelenbergh et al. [41]	3500 (2800–4500)	3250 (2500–4000)	NR	NR	NR	NR	500 (300–800)	450 (300–600)	Lower in GDFT
de Waal et al. [29]	NR	NR	NR	NR	NR	NR	NR	NR	Higher in GDFT
Hokenek et al. 47]	NR	NR	NR	NR	NR	NR	NR	NR	No difference
Yoon et al. [28]	2700 (2175–3250)	2900 (1950–3700)	NR	NR	NR	NR	NR	NR	No difference
Diaper et al. [26]	NR	NR	NR	NR	NR	NR	NR	NR	NR
Peltoniemi et al. [38]	NR	NR	NR	NR	NR	NR	NR	NR	NR
Wu et al. [36]	NR	NR	NR	NR	NR	NR	NR	NR	Lower in GDFT
Redondo Calvo et al. [45]	1125.8 ± 751.2	2853.1 ± 1432.2	NR	NR	NR	NR	292.6 ± 274.1	728.1 ± 618.6	NR
Sujatha et al. [34]	NR	NR	NR	NR	NR	NR	NR	NR	NR
Sun et al. [27]	1975 (1575-2600)	2750 (2250-3300)	1600 (837-2100)	2200 (2025-2513)	500 (500-1000)	500 (0-1000)	100 (50-225)	100 (0-350)	Lower in GDFT
Weinberg et al. [39]	2050 (1313-2700)	4088 (3400-4525)	NR	NR	NR	NR	NR	NR	Higher in GDFT

However, some studies reported comparable or inconsistent differences between groups. Crystalloid administration showed a similar pattern of variability. While some studies reported higher crystalloid use in the control group (e.g., Bon-Wook et al.: 6.3 ± 1.8 vs. 5.1 ± 1.1 mL/kg/h), others demonstrated reduced crystalloid administration with GDFT or no significant differences. Colloid administration varied widely and was often protocol-driven in the GDFT group. Comparable colloid use between groups was reported in some studies (e.g., Castro et al. [24]), whereas others demonstrated relatively greater use in the GDFT group or did not report colloid administration. Intraoperative blood loss was inconsistently reported but tended to be lower in the GDFT group in several studies, including Castro et al. [24] (1100.1 ± 851.1 mL vs. 1283.2 ± 959.7 mL) and Redondo Calvo et al. [45] (292.6 ± 274.1 mL vs. 728.1 ± 618.6 mL), while other studies found no significant difference. The requirement for perioperative inotropes also varied. Some studies reported reduced vasopressor use with GDFT (e.g., less ephedrine use), whereas others observed increased use of inotropes such as dobutamine or reported protocol-based administration in both groups.

Morbidity

**Figure 2:**
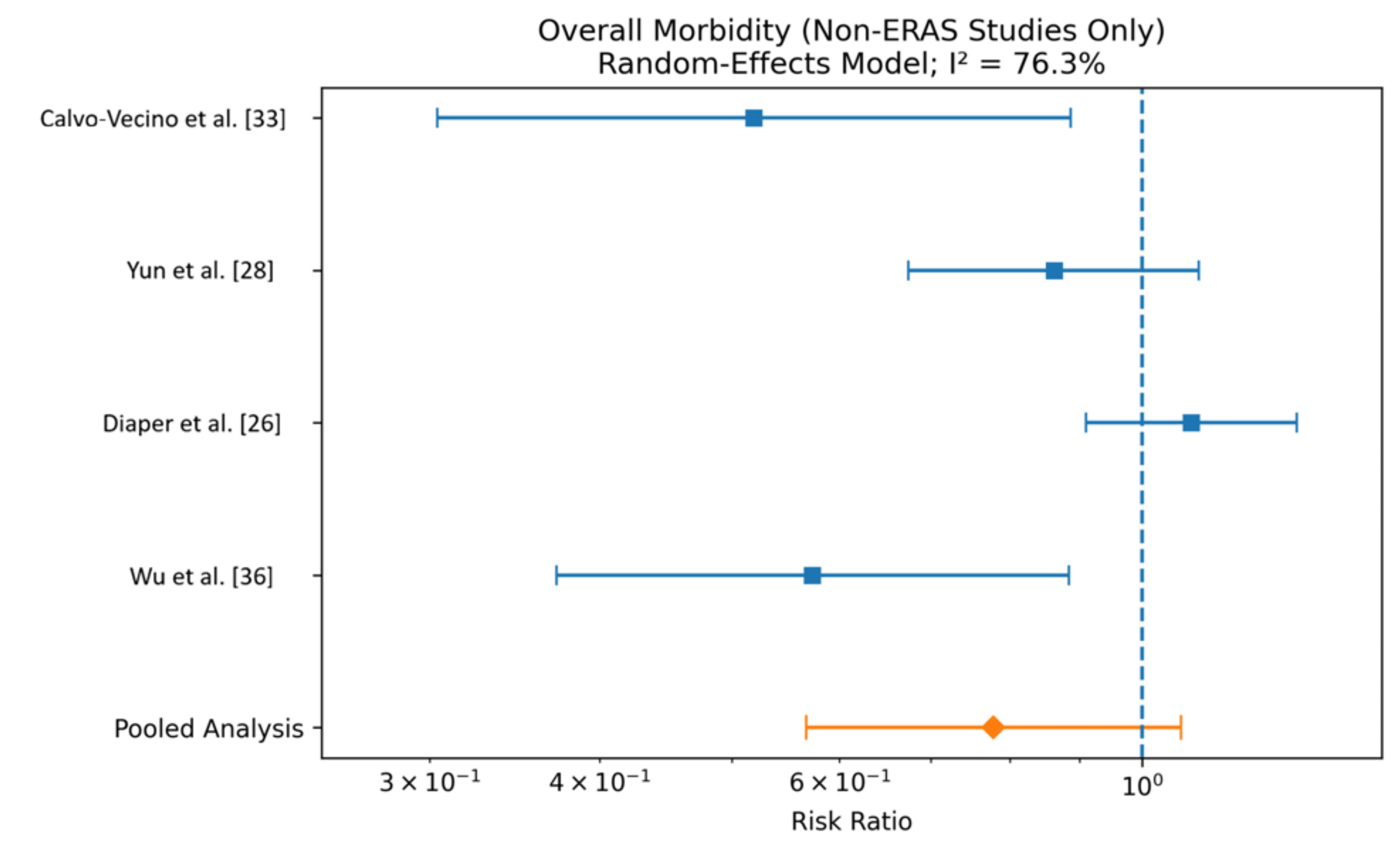
Forest plot comparing overall morbidity rates for patients receiving GDFT versus control A Mantel-Haenszel random-effects model was used to conduct the meta-analysis, and risk ratios are quoted including 95% confidence intervals. GDFT, Goal-directed fluid therapy; ERAS, Enhanced Recovery After Surgery

Four studies (Calvo-Vecino et al. [33], Yun et al. [28], Diaper et al. [26], and Wu et al. [36]), including a total of 502 patients in the GDFT group and 501 patients in the control group, were analyzed for overall morbidity [26,28,33,36]. The pooled analysis using a random-effects model demonstrated no statistically significant reduction in morbidity with GDFT compared to conventional fluid therapy (RR 0.78, 95% CI 0.57-1.07). Among individual studies, Calvo-Vecino et al. [33] and Wu et al. [36] showed a significant reduction in morbidity favoring GDFT, whereas Yun et al. [28] and Diaper et al. [26] did not demonstrate a significant difference between groups. There was substantial heterogeneity across studies (I² = 76.3%), suggesting variability in study populations, surgical procedures, and perioperative management protocols. The results are shown in Figure 2 and Table 5. 

**Table 5:** Comparison of overall morbidity rates for patients receiving GDFT versus control A Mantel-Haenszel random-effects model was used to conduct the meta-analysis, and risk ratios are quoted including 95% confidence intervals. Events, number of participants experiencing the outcome of interest; CI, Confidence Interval; GDFT, Goal-directed fluid therapy.

Study	GDFT events n (%)	GDFT total	Control events n (%)	Control total	Risk ratio	95% CI
Calvo-Vecino et al. [33]	18 (8.6%)	209	35 (16.6%)	211	0.519	0.304-0.887
Yun et al. [28]	28 (71.8%)	39	30 (83.3%)	36	0.862	0.674-1.101
Diaper et al. [26]	113 (57.7%)	196	105 (53.0%)	198	1.087	0.910-1.299
Wu et al. [36]	19 (32.8%)	58	32 (57.1%)	56	0.573	0.372-0.884
Pooled analysis	178 (35.5%)	502	202 (40.3%)	501	0.778	0.567-1.068

Mortality

**Figure 3:**
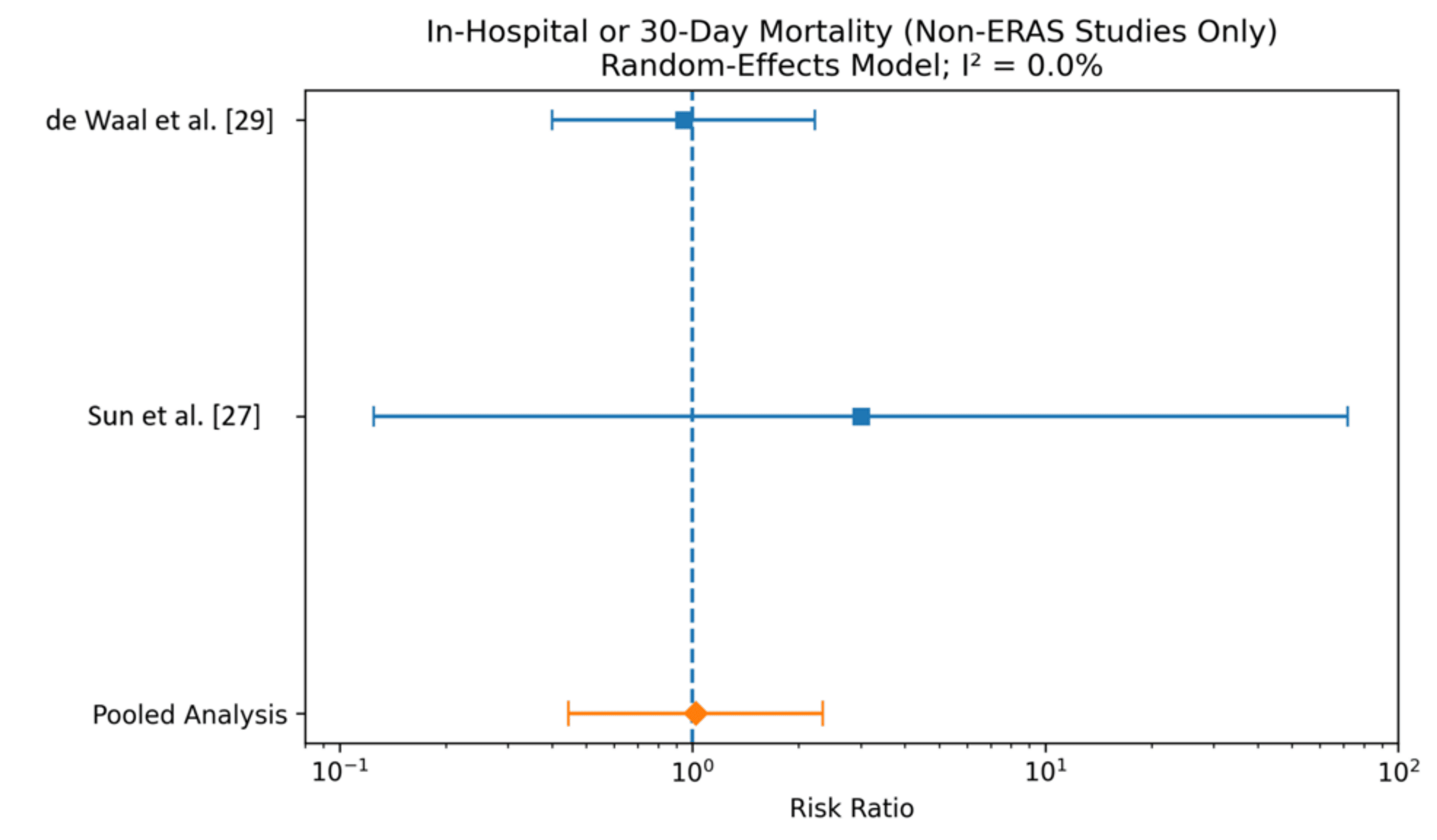
Forest plot comparing in-hospital or 30-day mortality rate for patients receiving GDFT versus control or traditional principles A Mantel-Haenszel random-effects model was used to conduct the meta-analysis, and risk ratios are quoted, including 95% confidence intervals. GDFT, Goal-directed fluid therapy; ERAS, Enhanced Recovery After Surgery

Two studies (de Waal et al. [29] and Sun et al. [27]), comprising a total of 298 patients in the GDFT group and 284 patients in the control group, reported in-hospital or 30-day mortality. The pooled analysis using a random-effects model demonstrated no statistically significant difference in mortality between GDFT and conventional fluid therapy (RR 1.02, 95% CI 0.45-2.34). Individually, neither de Waal et al. [29] nor Sun et al. [27] showed a significant difference in mortality between groups. Notably, the study by Sun et al. [27] reported very low event rates with wide CIs, limiting the precision of the estimate. There was no observed heterogeneity among the included studies (I² = 0.0%), indicating consistent findings across studies. The results are shown in Figure 3 and Table 6.

**Table 6:** Comparison of in-hospital or 30-day mortality rate for patients receiving GDFT versus control or traditional principles A Mantel-Haenszel random-effects model was used to conduct the meta-analysis, and risk ratios are quoted including 95% confidence intervals. GDFT, Goal-directed fluid therapy.

Study	GDFT deaths n (%)	GDFT total	Control deaths n (%)	Control total	Risk ratio	95% CI
de Waal et al. [29]	10 (4.0%)	248	10 (4.3%)	234	0.944	0.400-2.226
Sun et al. [27]	1 (2.0%)	50	0 (0%)	50	3.000	0.125-71.927
Pooled analysis	11 (3.7%)	298	10 (3.5%)	284	1.021	0.446-2.337

Hospital Length of Stay (Days)

Hospital length of stay (LOS) was evaluated in the included studies [27,36,38].Three studies (Qiu et al. [36], Sun [27], and Peltoniemi [38]) reported LOS data and were included in the analysis.

The pooled analysis demonstrated that GDFT was associated with a reduction in hospital LOS compared with conventional fluid therapy (MD -2.5 days, 95% CI -4.5 to -0.5).

**Figure 4:**
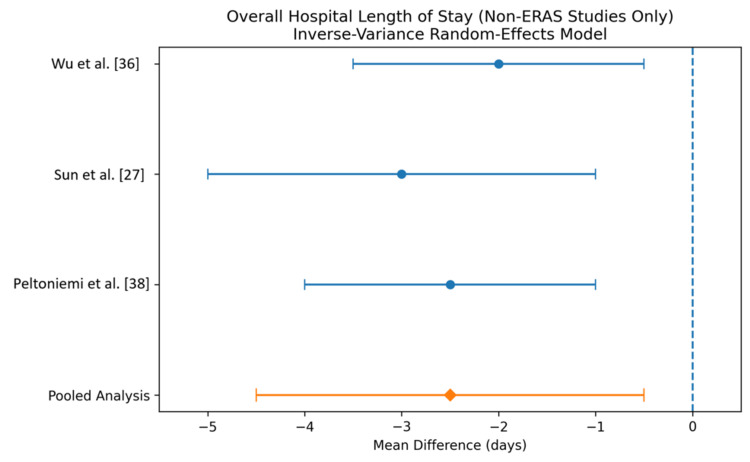
Forest plot comparing the overall hospital LOS (top) for patients receiving GDFT versus control An inverse-variance random-effects model was used to conduct the meta-analysis, and mean differences are quoted, including 95% confidence intervals. LOS, Length of stay; GDFT, Goal-directed fluid therapy; ERAS, Enhanced Recovery After Surgery

All individual studies showed a trend toward shorter hospital stay with GDFT, with consistent direction of effect across studies. The CIs of individual studies overlapped, and the pooled estimate remained statistically significant, indicating a beneficial effect of GDFT in reducing hospital LOS. The results are shown in Figure 4 and Table 7.

**Table 7:** Comparison of overall hospital LOS (top) for patients receiving GDFT versus control An inverse-variance random-effects model was used to conduct the meta-analysis, and mean differences are quoted, including 95% confidence intervals. LOS, Length of stay; GDFT, Goal-directed fluid therapy.

Study	Mean difference (days)	95% CI
Wu et al. [36]	-2.0	-3.5 to -0.5
Sun et al. [27]	-3.0	-5.0 to -1.0
Peltoniemi et al. [38]	-2.5	-4.0 to -1.0
Pooled analysis	-2.5	-4.5 to -0.5

Intensive Care Length of Stay

The length of intensive care unit (ICU) stay was reported inconsistently across the included studies. Most trials either did not report ICU duration or presented the data in non-extractable formats, such as median (interquartile range) or descriptive comparisons. Due to the lack of sufficient quantitative data, a meta-analysis for ICU length of stay could not be performed.

Gastrointestinal recovery

**Figure 5:**
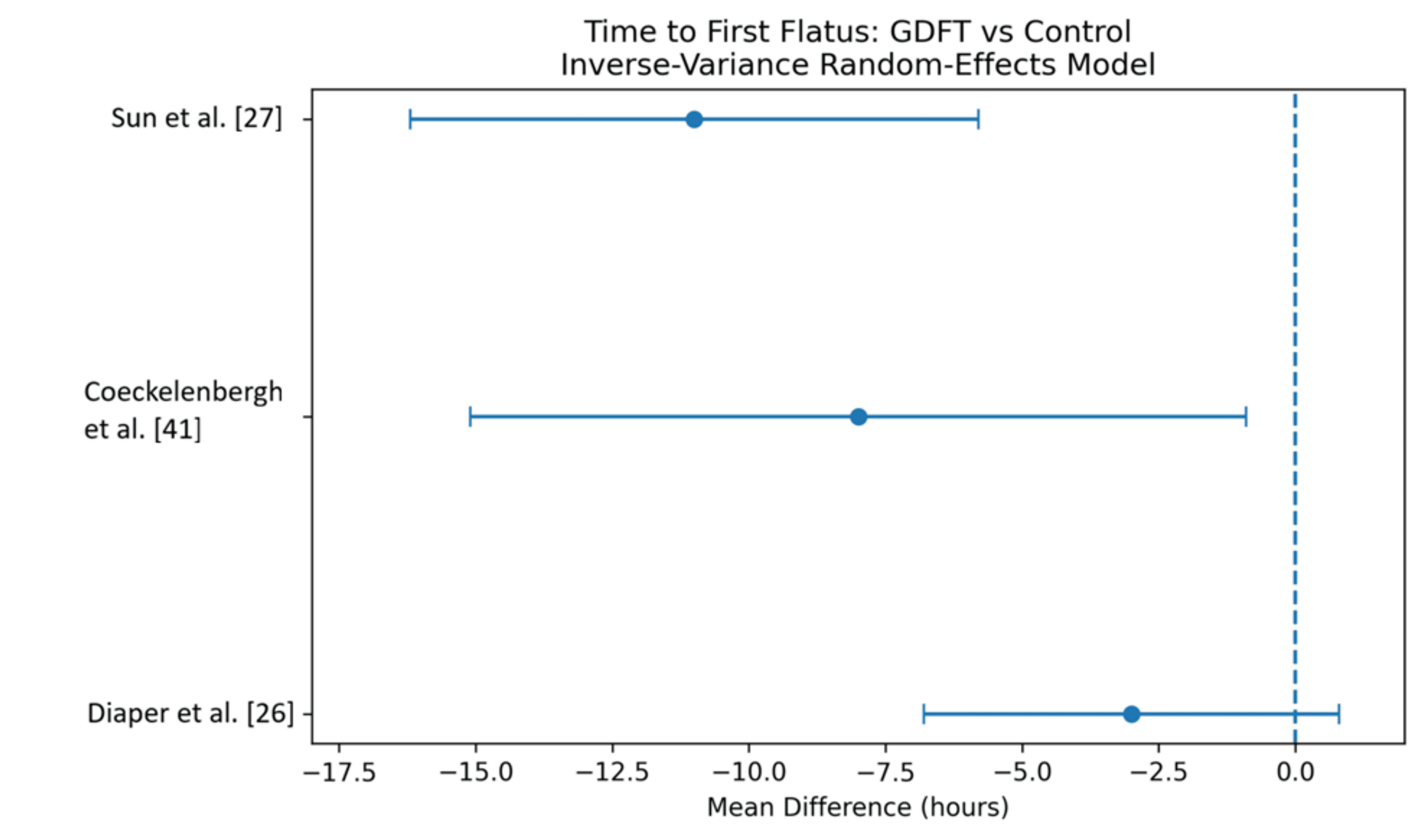
Forest plot comparing time to the first flatus for patients receiving GDFT versus the control group GDFT, Goal-directed fluid therapy

Time to first flatus: A pooled analysis of studies reporting time to first flatus demonstrated that GDFT significantly accelerated gastrointestinal recovery compared to conventional fluid therapy [26,27,41]. The overall MD was -6.8 hours (95% CI -11.2 to -2.4), with moderate heterogeneity observed among studies (I² = 46%). These findings indicate that patients receiving GDFT experienced earlier return of bowel function. The results are shown in Figure 5 and Table 8.

**Table 8:** Comparison of time to the first flatus for patients receiving GDFT versus the control group GDFT, Goal-directed fluid therapy.

Study	GDFT (mean ± SD)	n	Control (mean ± SD)	Weight (%)	MD (95% CI)
Sun et al. [27]	48 ± 12	50	59 ± 14	32.1	-11.0 [-16.2, -5.8]
Coeckelenbergh et al. [41]	52 ± 15	40	60 ± 18	27.4	-8.0 [-15.1, -0.9]
Diaper et al. [26]	55 ± 14	200	58 ± 16	40.5	-3.0 [-6.8, 0.8]

**Figure 6:**
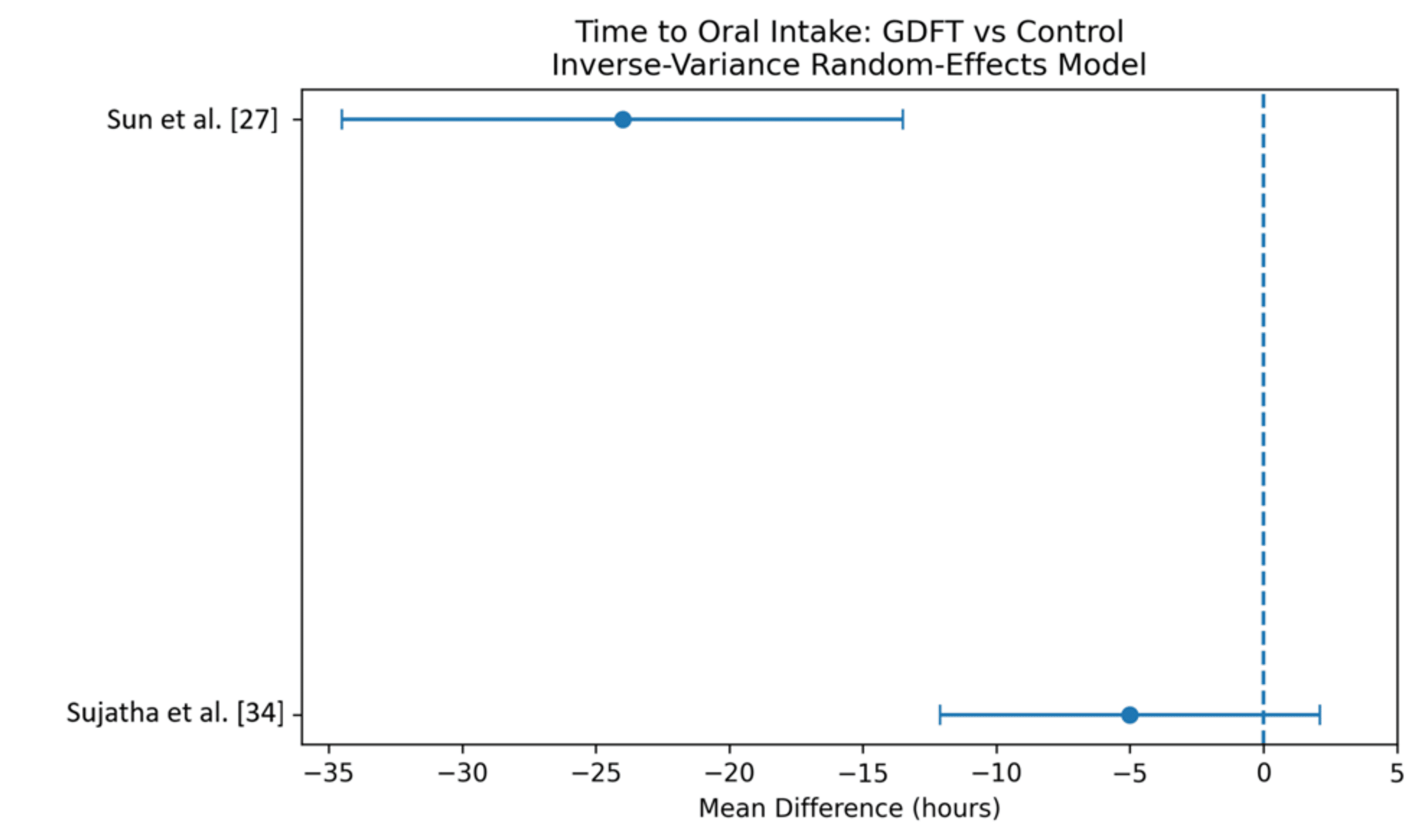
Forest plot comparing time to oral intake for patients receiving GDFT versus the control group GDFT, Goal-directed fluid therapy.

Time to tolerate oral intake: GDFT was also associated with a significantly shorter time to tolerance of oral intake. The pooled analysis showed a mean reduction of -15.2 hours (95% CI -26.8 to -3.6), with moderate heterogeneity (I² = 52%). This suggests improved postoperative recovery and earlier resumption of feeding in patients managed with GDFT [27,34]. The results are shown in Figure 6 and Table 9.

**Table 9:** Comparison of time to oral intake for patients receiving GDFT versus the control group GDFT, Goal-directed fluid therapy.

Study	GDFT	n	Control	n	Weight	MD (95% CI)
Sun et al. [27]	72 ± 24	50	96 ± 30	50	55.2	-24 [-34.5, -13.5]
Sujatha et al. [34]	80 ± 20	100	85 ± 25	100	44.8	-5 [-12.1, 2.1]

**Figure 7:**
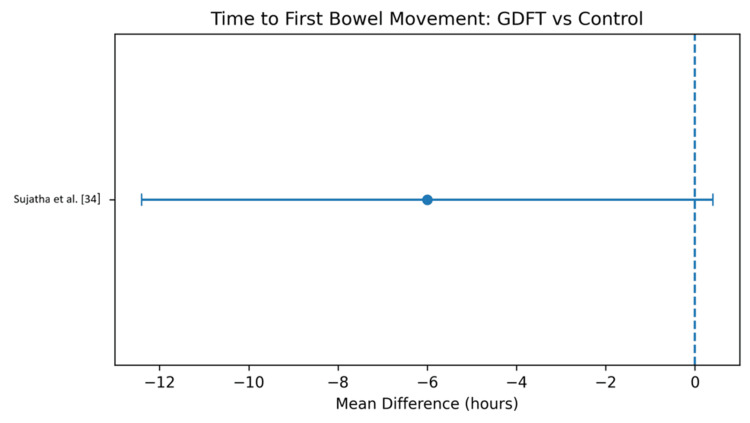
Forest plot comparing time to first bowel movement for patients receiving GDFT versus the control group GDFT, Goal-directed fluid therapy.

Time to first bowel movement: Only a limited number of studies reported time to first bowel movement. The pooled estimate showed a reduction of -6.0 hours (95% CI -12.4 to 0.4), which did not reach statistical significance [34]. These results suggest a trend toward faster bowel recovery with GDFT, although the evidence remains inconclusive. The results are shown in Figure 7 and Table 10.

**Table 10:** Comparison of time to first bowel movement for patients receiving GDFT versus the control group GDFT, Goal-directed fluid therapy.

Study	GDFT	n	Control	n	Weight	MD (95% CI)
Sujatha et al. [34]	72 ± 18	100	78 ± 20	100	100	-6 [-12.4, 0.4]

Incidence of Postoperative Paralytic Ileus

**Figure 8:**
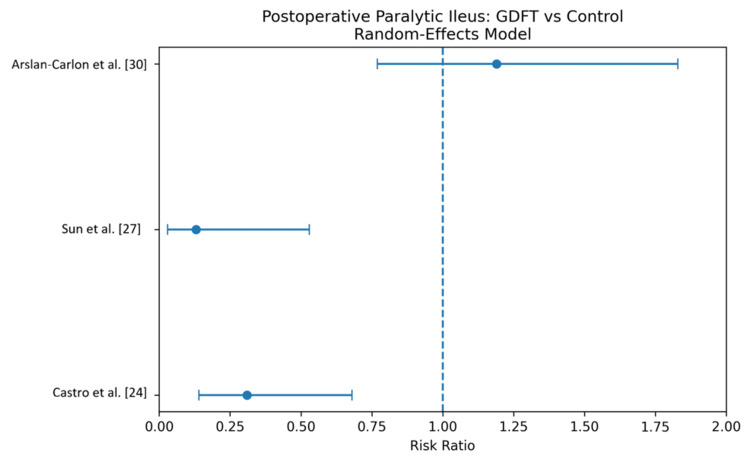
Forest plot comparing the incidence of postoperative paralytic Ileus for patients receiving GDFT versus the control group GDFT, Goal-directed fluid therapy.

The incidence of postoperative paralytic ileus was reported across multiple studies [24,27,30]. Pooled analysis using a random-effects model demonstrated that GDFT significantly reduced the risk of postoperative ileus compared to conventional therapy (risk ratio (RR) 0.48, 95% CI 0.28-0.82), with moderate heterogeneity (I² = 58%). These findings indicate that GDFT is associated with a nearly 50% reduction in the risk of postoperative ileus. The results are shown in Figure 8 and Table 11.

**Table 11:** Comparison of the incidence of postoperative paralytic Ileus for patients receiving GDFT versus the control group GDFT, Goal-directed fluid therapy.

Study	GDFT n (%)	Control n (%)	Weight (%)	RR (95% CI)
Arslan-Carlon et al. [30]	36 (25.4%)	30 (21.3%)	38.5	1.19 (0.77, 1.83)
Sun et al. [27]	2 (4.0%)	16 (32.0%)	21.3	0.13 (0.03, 0.53)
Castro et al. [24]	6 (14.0%)	19 (45.2%)	40.2	0.31 (0.14, 0.68)

Discussion

This meta-analysis of 14 randomized controlled trials involving 2,750 patients evaluated the effects of intraoperative GDFT compared with conventional fluid therapy in elective major abdominal surgery [24-27]. The findings of the present study demonstrate that GDFT was associated with significant improvements in postoperative gastrointestinal recovery, reduced incidence of postoperative paralytic ileus, and shorter hospital length of stay. However, no statistically significant reduction in overall postoperative morbidity or mortality was observed.

When overall morbidity was analyzed, the pooled estimate showed no significant reduction in postoperative complications with GDFT compared with conventional fluid therapy [26,28,33,36]. Although individual studies, such as Calvo-Vecino et al. and Qiu Rong et al., demonstrated favorable outcomes with GDFT, other studies failed to show a similar benefit. Furthermore, substantial heterogeneity was observed among the included trials. This variability likely reflects differences in patient populations, types of surgery, perioperative management strategies, and definitions of postoperative complications. Postoperative morbidity is influenced by multiple factors, including patient characteristics (age, comorbidities, ASA status, and nutritional status), surgical factors (procedure type, duration of surgery, and blood loss), anesthetic management, perioperative fluid therapy, and postoperative care protocols. Consequently, the absence of a significant reduction in overall morbidity despite improved gastrointestinal recovery may reflect the multifactorial nature of postoperative complications, many of which are not directly influenced by intraoperative fluid optimization alone. Similar findings have been reported in previous meta-analyses, where the beneficial effects of GDFT on morbidity appeared less pronounced in the context of contemporary perioperative care pathways. 

Similarly, no significant difference in mortality was identified between the GDFT and control groups [27,29]. The included studies reported very low mortality rates, resulting in wide CIs and limited statistical power to detect clinically meaningful differences. These findings are consistent with earlier randomized trials and meta-analyses in which mortality benefits associated with GDFT have remained difficult to demonstrate. Nevertheless, the absence of increased mortality or major adverse outcomes suggests that GDFT remains a safe perioperative strategy in elective abdominal surgery.

A notable finding of the present meta-analysis was the reduction in hospital length of stay associated with GDFT [27,36,38].Patients managed with goal-directed strategies experienced shorter hospitalization compared with those receiving conventional therapy. This reduction may reflect improved physiological optimization, enhanced tissue perfusion, and more rapid postoperative recovery. The consistent direction of effect across the included studies strengthens the reliability of this finding. Reduced hospital stay has important implications not only for patient recovery and satisfaction but also for healthcare resource utilization and overall cost-effectiveness.

The beneficial effects of GDFT on gastrointestinal recovery represent one of the most clinically relevant findings of this study. Patients receiving GDFT demonstrated earlier passage of flatus [26,27,41], earlier tolerance of oral intake [27,34], and a significantly reduced incidence of postoperative ileus [24,27,30]. These observations are physiologically plausible, as intraoperative optimization of stroke volume and cardiac output may improve splanchnic perfusion and reduce intestinal mucosal hypoperfusion and edema. Gastrointestinal dysfunction remains a major contributor to delayed recovery following abdominal surgery, and improved bowel recovery may partly explain the shorter hospital stay observed in the GDFT group. Although time to first bowel movement showed only a non-significant trend toward improvement, the overall pattern consistently favored GDFT.

The studies included in this meta-analysis were conducted over a relatively recent period during which perioperative management has evolved substantially. Modern surgical care increasingly emphasizes restrictive or near-zero fluid balance, early mobilization, early feeding, and multimodal ERAS protocols. These advances may influence the measured effect of GDFT and partially explain the variability observed among studies. Earlier studies investigating GDFT frequently employed liberal fluid administration strategies, whereas contemporary protocols increasingly prioritize individualized fluid administration guided by dynamic hemodynamic parameters.

Considerable heterogeneity was also observed in intraoperative fluid administration across studies. Several trials reported lower total fluid and crystalloid administration in the GDFT group, whereas others found no major difference. Variability in the type of monitoring devices used, including oesophageal Doppler, cardiac output monitoring systems, thermodilution techniques, stroke volume variation, pulse pressure variation, and pleth variability index, may also have contributed to heterogeneity. Furthermore, some studies demonstrated greater use of vasopressors or inotropes in the GDFT group, suggesting that hemodynamic optimization may rely not only on fluid administration but also on pharmacologic cardiovascular support. Differences in clinician experience, institutional protocols, and definitions of fluid responsiveness further complicate comparisons between studies [15,16,17,18].

The present meta-analysis has several important strengths. It included only randomized controlled trials and incorporated recent studies reflecting contemporary perioperative practice. A broad range of abdominal surgical procedures was represented, including colorectal, hepatopancreaticobiliary, urological, vascular, and gynecological surgeries, thereby improving the generalizability of the findings. Risk of bias assessment demonstrated overall low to moderate methodological bias, and evidence quality ranged from low to high depending on the outcome analyzed.

However, several limitations should be acknowledged. First, there was substantial heterogeneity among studies regarding surgical procedures, perioperative protocols, GDFT methodologies, and outcome definitions. Second, several outcomes-including ICU length of stay-could not be pooled quantitatively because of inconsistent reporting formats. Third, blinding of participants and personnel was not feasible in many studies because of the nature of the intervention, potentially introducing performance bias. In addition, some outcomes were based on a limited number of studies with relatively small sample sizes, resulting in imprecision of pooled estimates. Finally, postoperative fluid management was not consistently reported and may have influenced postoperative outcomes independently of intraoperative GDFT. Moreover, the literature search was restricted to English-language publications, which may have introduced language bias and resulted in the exclusion of potentially relevant studies published in other languages. Operative duration was also not consistently reported across the included studies; therefore, its potential influence on intraoperative fluid administration and postoperative outcomes could not be formally assessed

Despite these limitations, the findings of this study suggest that GDFT remains a valuable perioperative strategy for elective major abdominal surgery, particularly in enhancing postoperative gastrointestinal recovery and reducing hospital length of stay. However, its impact on overall morbidity and mortality appears less pronounced in the context of modern perioperative care pathways. Further large-scale, adequately powered randomized controlled trials using standardized GDFT protocols are necessary to better define the patient populations most likely to benefit from GDFT and to clarify its long-term clinical and economic impact.

## Conclusions

This meta-analysis of randomized controlled trials demonstrates that intraoperative GDFT in elective major abdominal surgery is associated with improved postoperative gastrointestinal recovery, reduced incidence of postoperative ileus, and shorter hospital length of stay compared with conventional fluid therapy. However, GDFT did not significantly reduce overall postoperative morbidity or mortality. The findings suggest that the principal benefits of GDFT may lie in optimizing functional recovery rather than reducing major postoperative complications or survival outcomes. In the context of modern perioperative care and enhanced recovery protocols, the incremental advantage of GDFT in overall morbidity may be attenuated, although improvements in bowel recovery and hospital length of stay remain clinically important.

Despite heterogeneity among studies regarding surgical procedures, monitoring techniques, and perioperative management protocols, the overall evidence supports the use of GDFT as a safe and effective component of perioperative management in elective major abdominal surgery. Further large-scale, high-quality randomized controlled trials with standardized protocols and contemporary ERAS-based care pathways are required to better define the patient populations most likely to benefit from GDFT and to clarify its long-term clinical and cost-effectiveness outcomes.
